# Increased risk of bone tumors after growth hormone treatment in childhood: A population‐based cohort study in France

**DOI:** 10.1002/cam4.1602

**Published:** 2018-06-14

**Authors:** Amélie Poidvin, Jean‐Claude Carel, Emmanuel Ecosse, Dominique Levy, Jean Michon, Joël Coste

**Affiliations:** ^1^ Assistance Publique‐Hôpitaux de Paris (AP‐HP) Hôpital Universitaire Robert‐Debré Department of Pediatric Endocrinology and Diabetology Centre de Référence des Maladies Endocriniennes Rares de la Croissance Paris France; ^2^ PROTECT INSERM Université Paris Diderot Sorbonne Paris Cité Paris France; ^3^ Assistance Publique‐Hôpitaux de Paris, Biostatistics and Epidemiology Unit Hôtel Dieu Paris France; ^4^ Institut Curie Paris France; ^5^ Université Paris Descartes Sorbonne Paris Cité Paris France

**Keywords:** Bone tumors, childhood, growth hormone treatment

## Abstract

The association between growth hormone (GH) treatment and cancer risk has not been thoroughly evaluated and there are questions about any increased risk of bone tumors. We examined cancer risk and especially bone tumor risk in a population‐based cohort study of 6874 patients treated with recombinant GH in France for isolated GH deficiency, short stature associated with low birth weight or length or idiopathic short stature. Adult mortality and morbidity data obtained from national databases and from questionnaires. Case ascertainment completeness was estimated with capture‐recapture methods. Standardized mortality and incidence ratios were calculated using national reference data. 111 875 person‐years of observation were analyzed and patients were followed for an average of 17.4 ± 5.3 years to a mean age of 28.4 ± 6.2 years. For cancer overall, mortality and incidence were not different from expected figures. Five patients developed bone tumors (chondrosarcoma, 1, Ewing sarcoma, 1, osteosarcoma, 3) of whom 3 died (Ewing sarcoma, 1, osteosarcoma, 2), whereas only 1.4 case and 0.6 deaths were expected: standardized mortality ratio, 5.0 and standardized incidence ratio from 3.5 to 3.8 accounting or not accounting for missed cases. Most patients received conventional doses of GH, although one patient with osteosarcoma had received high dose GH (60 μg/kg/d). This study confirms an increased risk of bone tumors but not overall cancer risk in subjects treated with GH in childhood for isolated GH deficiency or childhood short stature. Further work is needed to elucidate the mechanisms involved.

## 1 INTRODUCTION

The risk of malignancy after growth hormone treatment during childhood remains unclear. In experimental studies, growth hormone (GH) and insulin‐like growth factors (IGFs) have mitogenic, antiapoptotic, and proliferative properties.^1^, ^2^, ^3^ High levels of circulating IGF‐I have been shown to be associated with increased risks of several common cancers, including breast, prostate, and colorectal cancers 3, 4, 5 and GH itself could also play a direct role in carcinogenesis^6^, ^7^; notably, patients with acromegaly have consistently been found to have increased risks of several cancers, especially colorectal cancer.^8^ Also, patients with GH resistance and IGF‐I deficiency due to GH resistance have a significantly decreased risk of cancer.^9^, ^10^ The risk of several cancers is linked to height with taller people having a higher risk.^11^, ^12^, ^13^, ^14^, ^15^, ^16^, ^17^, ^18^

Few studies have addressed growth hormone therapy and cancer, however, and their systematic review by Deodati et al^19^ suggests that cancer mortality is not increased although cancer incidence is increased; these conclusions are not definitive, given the size and the methodology of the studies. An increased risk of second neoplasm in GH‐treated patients has also been reported, in particular for bone tumors.^20^

The Safety and Appropriateness of Growth hormone treatments in Europe (SAGhE) project is a multinational European study that aims to evaluate long‐term mortality and cancer morbidity in subjects who were treated with recombinant GH in childhood. A preliminary report in 2012 describing the French cohort of the SAGhE study indicated that all‐type cancer‐related mortality was not higher among those treated for idiopathic short stature or isolated GH deficiency than in the general population (standardized mortality ratio [SMR] 1.02, 95% CI 0.41‐2.09). Nevertheless, the bone tumor‐related mortality was high (SMR 5.00, 95% CI 1.01‐14.63).^21^ More recently, the European consortium published results for cancer mortality in all eight country cohorts and results for cancer incidence for five countries (not France, Germany, and Italy).^22^ In GH‐treated patients without previous cancer, there was no increased risk of cancer or cancer mortality overall but there was an excess risk of cancer mortality and incidence for bone cancer, an increased mortality by prostate cancer (1 case) and an increased incidence of bladder cancer.

The French SAGhE study data is particularly high quality, being based on a national register of GH‐treated children (the France Hypophyse register), and completed by data extracted from the Center for Epidemiology on Medical Causes of Death and from the French national health insurance information system. These data allow us to examine in more detail cancer morbidity and mortality in this population.

## 2 PATIENTS AND METHODS

### 2.1 Patients

**Figure 1:**
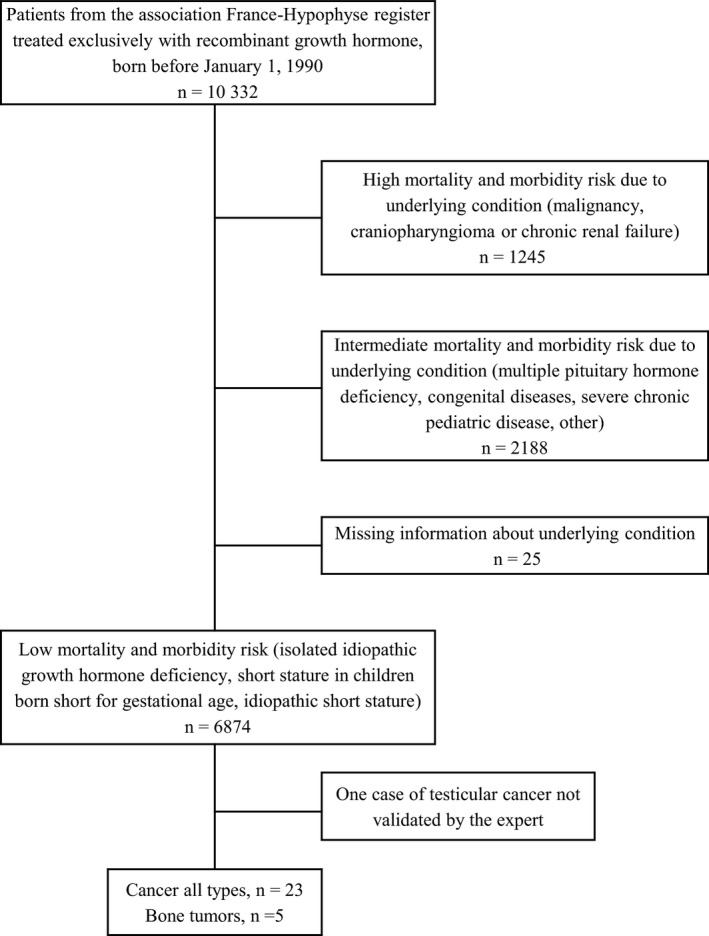
Flow chart of the SAGhE study in France and number of cancer cases identified in the low‐risk group

As described previously 21, 23, 24 we used the mandatory register of patients treated with GH in France [Association France‐Hypophyse] which was disbanded in 1996; we selected those patients had been treated exclusively with recombinant GH and who were born before 1 January 1990. Patients were assigned to three risk categories (low, medium, and high) for long‐term morbidity and mortality, based on the clinical condition leading to the initiation of GH treatment (Figure 1). Only low‐risk patients were included in this study, because their baseline risk of cancer is believed to be similar to or lower than that of the general population.

### 2.2 Data collected

#### 2.2.1 Childhood data

Data on patient characteristics, treatment, and growth progression in the France‐Hypophyse register were routinely collected at baseline and at regular follow‐up visits until 1996, and were obtained from pediatric endocrinologists. Additional follow‐up data on GH treatment were collected from clinical centers in 2008‐2010.

#### 2.2.2 Follow‐up data

Information on vital status was collected from the *Répertoire National d'Identification des Personnes Physiques* (http://www.insee.fr/fr/methodes) and the *Répertoire National Inter‐régimes de l'Assurance Maladie*. The cause of death indicated in death certificates was obtained from the French Center for Epidemiology on Medical Causes of Death (CépiDC, *Institut National de la Santé et de la Recherche Médicale*) and coded according to the 10th revision of the *International Classification of Diseases (ICD‐10)*.

Morbidity data were collected through a health questionnaire sent to all live patients (with a response rate of 46% after several reminders: the low response rate was expected, because questionnaires were sent to the parents’ address; there had been no prior contact between the researchers and the patients; and these patients generally do not feel “sick”). Data were also extracted using patient identifiers from the French national health insurance information system (*Système National d'Information Inter‐régimes de l'Assurance Maladie*; SNIIRAM); this system includes the French hospital discharge database (FHDD) also called *Programme de Médicalisation des Systèmes d'Information* from 1 January 2008 to 31 December 2010, and long‐lasting affection statements (LLA) which identify conditions with 100% reimbursement coverage (which include cancers). We set a censor date of 31 December 2010.

#### 2.2.3 Validation of events

Cancer events were validated using all medical and pathology reports obtained. Data about bone tumors were specifically reviewed by two oncologists specialized in these tumors (DL, JM).

### 2.3 Statistical analyses

The overall risk of cancer was assessed by calculating standardized incidence ratios (SIR), with adjustment for age and sex, using reference rates for all cancer combined from population‐based registries of cancer in France, centralized by the FRANCIM (*FRANce‐Cancer‐Incidence et Mortalité*) network, between 1985 and 2010.^25^ The risk of bone tumor was assessed using data provided by FRANCIM combined six general registries of bone tumors in France. The number of person‐years at risk was calculated for GH‐treated subjects, for each 5‐years age class, separately for men and women, from the date of first administration of GH to the date of cancer, death, loss to follow‐up, or 31 December 2010. The expected number of events was then calculated for GH‐treated subjects by multiplying the age‐ and sex‐specific incidence rates by the number of person‐years at risk. SIRs were estimated by dividing the number of observed events by the number of expected events. Significance tests and 95% confidence intervals (CI) for the SIR were calculated with Byar's approximation to the exact Poisson test and the exact Poisson limits. Standardized mortality ratios (SMR) and 95% CIs were calculated as reported previously.^21^

The CépiDC source for fatal events is exhaustive but the three sources (SNIIRAM — LLA, SNIIRAM — FHDD — and questionnaires) used to identify nonfatal events are not and therefore some events could have been missed by all sources. We used capture‐recapture methods 26, 27 to estimate the number of nonfatal events missed by all three sources. These methods are commonly used in epidemiology to estimate the completeness of ascertainment of disease registries and to estimate the number of cases that could have been missed by all sources of ascertainment. Thus, they allow improved accuracy of estimation of disease or event rates. Log‐linear modeling was used to adjust for source dependence by including the corresponding interaction term into the model,^23^ and the significance of the interaction was assessed using likelihood ratio statistics. A confidence interval (CI) for the estimated number of cases missed was computed by the profile likelihood method. The Akaike Information Criterion (AIC) was used for selection of the model. Two analyses were carried out: one with observed events, giving the crude SIR, the other including estimated events using the capture‐recapture method, giving the corrected SIR. Because of the small number of events for bone tumors, the ratio of estimated/observed nonfatal events for the whole cancer group was used to estimate the number of nonfatal events of bone tumors.

This study was approved by the *Comité Consultatif sur le Traitement de l'Information en Matière de Recherche dans le Domaine de la Santé* and the *Commission Nationale de l'Informatique et des Libertés* (the national data protection agency). The use of the *Registre National Inter‐Régimes de l'Assurance Maladie* was approved by a specific statute.

## 3 RESULTS

Most of the low‐risk mortality and morbidity group (n = 6874) were patients treated for the indication of isolated GH deficiency, based on GH stimulation tests (peak below 10 ng/mL) (n = 4600, 67%) (Table 1). In this group, there were over 111 000 person‐years at risk and the mean follow‐up between the beginning of GH treatment and the date of last follow‐up, event, or death was 17.4 years. The mean (±SD) treatment duration was 3.9 (±2.6) years, with mean doses slightly below the current recommendations for isolated GH deficiency.

**Table 1:** Main characteristics of patients and GH treatment for studied sample. N = 6874

Number of male patients (%)	4510 (66)
Indication for GH treatment. number (%)	
Isolated GH deficiency	
Maximum peak GH <3 μg/L	295 (4)
Maximum peak GH ≥3 μg/L and <7 μg/L	1557 (23)
Maximum peak GH ≥7 μg/L and <10 μg/L	2748 (40)
Missing value for maximum peak GH	516 (8)
Maximum peak GH ≥10 μg/L	
Neurosecretory dysfunction	547 (8)
Idiopathic short stature	868 (13)
Small for gestational age	343 (5)
Year of treatment start. number (%)	
1985‐1987	506 (7.4)
1988‐1990	2470 (36)
1991‐1993	2362 (34)
1994‐1996	1536 (22.6)
Birth length (SDS for gestational age)	−1.2 ± 1.2 (n = 4875)
Birth weight (SDS for gestational age)	−0.6 ± 1.2 (n = 5130)
Children born small for gestational age (birth weight or length ≤−2 SDS for gestational age)	
Yes	1298 (19)
No	3864 (56)
Missing data	1712 (25)
Chronological age at start of treatment (y)	11.0 ± 3.4 (n = 6874)
Height at start of treatment (SDS)	−2.7 ± 0.8 (n = 6285)
Weight at start of treatment (SDS)	−1.6 ± 0.9 (n = 6242)
Mean dose (μg/kg/d)	24.5 ± 12.3 (n = 6212)
Treatment duration (y)	3.9 ± 2.6 (n = 6380)
Chronological age at end of treatment (y)	15.1 ± 2.7 (n = 6380)
Person‐years of observation (n)	111 875
Chronological age at the time of census or event or death (y)	28.4 ± 6.2
Duration of follow‐up from start of GH to time of census or event or deaths (y)	17.4 ± 5.3 (n = 6616)

Mean ± SD or n (%) are shown. GH, growth hormone; SDS, SD score.

Twenty‐four cancer events were identified in this group through the different sources, including one case which was not validated (Table 2). The most common cancers were bone tumors (n = 5), lymphoma (n = 4), and acute leukemia (n = 3). Overall, the patients were treated with conventional doses of GH (mean 23.4 μg/kg/d) with only one patient receiving high GH doses (60 μg/kg/d). None of the patients presented particularly severe short stature at the beginning of treatment or exhibited substantial height gain during treatment. Eight patients, including two with osteosarcoma and one with Ewing sarcoma, died at a mean age of 17.3 years.

**Table 2:** Clinical characteristics and GH treatments in the 23 patients who developed cancer after GH treatment

#	Type of cancer	Died (Y/N)	Age at event or death (y)^a^	Sex	Diagnosis leading to GH treatment	Age at start of GH treatment (y)	Height (SD) before treatment	GH treatment duration (y)	Height (SD) at the end of treatment	Adult height (SD)	Mean GH dose (μg/kg/d)	Source of information for cancer
1	Acute leukemia	Y	12.9	F	GHDI	11.1	‐	1.4	‐	‐	‐	CépiDC
2	Acute leukemia	Y	14.8	M	GHDI	12.7	−2.1	0.8	−1.8	‐	23.5	CépiDC
3	Acute leukemia	Y	16.5	F	GHDI	9.2	‐	1.9	‐	‐	‐	CépiDC
4	Lymphoma	N	29.0	F	GHDI	12.0	−2.5	3.3	−2.1	−1.5	19.2	Questionnaire
5	Lymphoma	N	11.5	M	GHDI	7.0	−1.9	3.7	−1.0	−2.1	17.1	Questionnaire
6	Lymphoma	N	24.4	M	GHDI	9.9	−2.2	5.6	−0.6	‐	23.4	LLA. Questionnaire
7	Lymphoma	N	22.4	M	GHDI	8.6	−2.0	4.0	−1.9	‐	21.1	LLA. FHDD
9	Ewing sarcoma	Y	17.3	M	GHDI	10.3	−3.5	5.1	−2.4	‐	24.4	CépiDC
8	Osteosarcoma	Y	14.4	M	SGA	8.5	−3.6	3.0	−2.4	‐	60.0	CépiDC
10	Osteosarcoma	Y	20.2	F	GHDI	4.5	−2.7	8.2	‐	‐	26.6	CépiDC
11	Osteosarcoma	N	7.4	M	GHDI	6.1	−3.3	1.1	−2.9	−3.6	24.1	LLA. Questionnaire
12	Chondrosarcoma	N	21.8	M	GHDI	15.8	−1.3	1.2	−0.7	−0.7	26.1	Questionnaire
13	Malignant brain tumor	N	18.2	M	GHDI	2.4	‐	9.0	‐	‐	‐	LLA
14	Melanoma	Y	28.1	M	GHDI	11.1	−2.6	6.1	−1.8	−2.0	17.3	CépiDC
15	Melanoma	N	31.5	F	GHDI	15.1	‐	1.9	‐	−1.5	‐	Questionnaire
16	Nasopharyngeal carcinoma	Y	19.3	M	GHDI	11.2	‐	1.4	‐	‐	‐	CépiDC
17	Malignant tumor of the mouth	N	26.4	M	GHDI	8.5	−2.0	7.1	−0.6	−0.9	21.5	LLA. FHDD
18	Malignant tumor of the kidney	N	29.5	F	GHDI	9.6	‐	2.4	‐	−2.7	‐	LLA. FHDD. Questionnaire
19	Malignant tumor of the testis	N	31.6	M	GHDI	12.2	‐	2.0	−1.8	−0.9	15.8	LLA. FHDD. Questionnaire
20	Malignant tumor of the testis	N	15.0	M	GHDI	12.7	−1.9	3.5	−1.3		27.9	Questionnaire
21	Sweat gland carcinoma	N	32.6	F	GHDI	13.9	−2.7	2.5	−2.2	−2.3	28.0	Questionnaire
22	Pulmonary carcinoma	N	26.9	M	GHDI	8.0	−1.7	3.7	−1.2	−1.7	17.2	FHDD. Questionnaire
23	Malignant pancreatic tumor	N	19.4	M	GHDI	8.2	−2.2	6.6	−0.5	−1.1	16.7	LLA. FHDD. Questionnaire

LLA, long‐lasting affection; FHDD, French hospital discharge database; F, female; M, male; GHDI, idiopathic growth hormone deficiency; SGA, Born small for gestational age.

a Age at event was used for live patients, and age at death was used for those who died.

Bone tumor characteristics are shown in Table 3. There were three osteosarcomas occurring 1.1, 2.9, and 15 years after the start of GH treatment; the diagnosis of cancer led to the interruption of GH treatment in the first two cases. Two of the patients with osteosarcoma died, 6 months‐3 years, after diagnosis. There was one fatal case of Ewing sarcoma, with bone and bone marrow metastases, 5.1 years after GH treatment initiation and 1.5 years after GH treatment interruption. There was one case of chondrosarcoma, 6 years after GH treatment initiation and 4.8 years after GH treatment interruption, and this patient is alive 8 years after surgery. One of the patients with osteosarcoma had received high GH doses (60 μg/kg/d).

**Table 3:** Clinical characteristics, treatment and evolution related to GH and bone tumors in the 5 patients with bone tumors

#	Type of cancer	Localization	Age at diagnosis of cancer (y)	Delay between start of GH and cancer diagnosis (y)	Clinical manifestations of cancer	Metastasis	Metastasis localization	Initial cancer treatment	Complete remission	Relapse	Treatment of cancer relapse	Died	Medical and pathology reports available
8	Ewing sarcoma	Sacrum	15.9	6.6	Dysuria, constipation, pain	Y	Bone and bone marrow	Chemotherapy, surgery and radiotherapy	Y	Y	Oral chemotherapy (VP16)	Y	Y
9	Osteosarcoma	Left lower tibia	11.5	2.9	Pain	N		Chemotherapy, surgery and radiotherapy	Y	Y	CHD	Y	Y
10	Osteosarcoma	Iliac wing	19.5	15	Pain	N		Chemotherapy and radiotherapy (no surgery because of deep vein thrombosis)	N			Y	Y
11	Osteosarcoma	Lower end of the right femur	7.4	1.1	U	U		U	U			N	N
12	Chondrosarcoma	Inter‐tibio‐fibular	21.8	6	U	N		Surgery	Y	N		N	Y

Y, Yes; N, No; U, Unknown.

SIRs and SMRs for all cancers and bone tumors are presented in Table 4. The risk of cancer overall was not different from expected values in patients treated with GH (SIR 0.7, 95% CI 0.5‐1.1). The risk of bone tumor was significantly higher than expected in this population (SIR 3.5, 95% CI 1.1‐8.1). After capture‐recapture analysis (Table 4 and S1), the estimated number of missed cases of cancer overall was 3.5 (95% CI 0.8‐15.3) and the corrected SIR were 0.8 (95% CI 0.5‐1.2) for all cancers and 3.8 (95% CI 1.3‐8.6) for bone tumors. The SMR was 1.0 (95% CI 0.4‐2.1) for cancer overall and 5.0 (95% CI 1.0‐14.6) for bone tumors.

**Table 4:** Crude SMR and SIR and corrected SIR for all‐type cancer and bone tumors in young adults treated with growth hormone in childhood

	Crude						Corrected		
	Observed deaths (n)	Expected deaths (n)	Crude SMR (95% CI)	Observed events (n)	Expected events (n)	Crude SIR (CI 95%)	Estimated events (n)	Expected events (n)	Corrected SIR (CI 95%)
All cancers	7	6.9	1.0 (0.4‐2.1)	23	32.3	0.7 (0.5‐1.1)	26.5	32.3	0.8 (0.5‐1.2)
Bone tumors	3	0.6	**5.0 (1.0‐14.6)**	5	1.4	**3.5 (1.1‐8.1)**	5.5	1.4	**3.8 (1.3‐8.6)**

Bold values indicate statistical significance.

## 4 DISCUSSION

We report an analysis using the largest national population‐based register of patients treated with GH during childhood. Our study shows a 3.5‐3.8‐fold higher incidence of and a 5‐fold higher mortality from bone tumors in patients treated with GH for idiopathic isolated GH deficiency, idiopathic short stature, or short stature in children born short for gestational age, than in the general population. This raises issues about the mechanism, possible confounders, and causal effect of GH treatment. In contrast, the incidence and mortality of cancer overall were not different from those in the general population.

Growth hormone and IGF‐I act on bone and cartilage, specifically on chondrocytes and osteoblasts 28, 29, 30 and the GH receptor is strongly expressed on these cell types. Osteosarcomas occur preferentially in rapidly growing bones, during the rapid bone growth of puberty and in tall adolescents (patients are 0.3 SD taller than the general population).^31^, ^32^, ^33^ Observations of childhood cancer survivors indicate that GH treatment is associated with a large increase in the risk of osteosarcoma^20^: 15% of second neoplasms in this population are osteosarcomas.^34^, ^35^ The European SAGhE study has analyzed cancer morbidity using data from five countries (excluding France, due to methodological issues) 22 and also found an excess risk of bone tumors (SIR of 4.1) albeit without a clear dose‐effect relationship. Thus, evidence is growing that GH treatment may have a biological effect on bone tumor initiation, promotion, and/or progression.

The risk of cancer overall in our population of GH‐treated subjects with an SIR of 0.7‐0.8 and an SMR of 1.0 tended to be lower than that in the general population. This is consistent with the results of the meta‐analysis by Deodati et al^19^ — who provided summary tables of studies of cancer incidence and mortality following GH administration, with or without a cancer history — and those of the European SAGhE study.^22^ It is also consistent with the evidence that cancer incidence is lower in short stature than general populations, with hazard ratios applied to our cohort giving an estimated SIR of 0.8‐0.9.^11^, ^12^, ^13^, ^14^, ^15^, ^16^, ^17^, ^18^ Our observations for bone tumors are therefore even more striking, as the SIR would be expected to be lower than normal in a population of short individuals.

Our study has several potential limitations. First, noncompleteness of the sources of ascertainment of cancer was a limitation for the analysis of the incidence of cancer. We addressed this limitation by using a capture‐recapture method which gave reassuring results on the validity of our crude analysis. Capture‐recapture methods have been successfully applied in a wide range of epidemiologic fields to estimate the completeness of ascertainment of registries. Such estimation of the potential number of cases (sometimes high) missed by all sources of ascertainment improves the accuracy of assessment of disease rates.^27^ The second limitation is that the numbers of events and especially of fatal events (both in the study cohort and in the reference registries) are small such that the CIs, in particular CIs of SMRs, were wide. The number of events was too small to allow testing for a relationship between the dose of GH treatment and the incidence of bone tumors. Another limitation was that we were unable to include a comparable reference group of short‐stature individuals not treated with GH. Also there is the issue of whether patients with a genetic predisposition to bone tumors were inadvertently included in our low‐risk group of patients; this issue is important because retrospective genetic analysis of families was not possible in our study for legal and ethical reasons. Several genetic syndromes, some of them associated with short stature (Bloom, Rothmund‐Thompson, Werner, hereditary retinoblastoma) are risk factors for osteosarcomas 32 but would not commonly be misdiagnosed for idiopathic isolated GH deficiency. Li‐Fraumeni syndrome, by contrast, could go clinically unnoticed if questions about familial history of tumor in childhood or young adults are not asked before beginning GH treatment but is generally not associated with short stature. Mild Fanconi anemia, which could be misdiagnosed for some time in short‐stature children, does not seem to be associated with an increased of bone tumors, contrary to other forms of malignancy (leukemias, myelodysplastic syndromes, solid tumors of head, neck, skin, gastrointestinal tract, and genitourinary tract).^36^ These rare genetic syndromes, none of which are strongly associated with both the risk of short stature and the risk of bone tumor, are therefore unlikely to confound our results. Finally, the reference data for bone tumors we used was based on six regional registries and not national data.

In conclusion, our analyses support the view that subjects treated with GH in childhood for isolated GH deficiency or childhood short stature are at increased risk of bone tumors but not of cancer overall. A causal relationship between GH treatment and bone tumors is biologically plausible but the mechanisms remain to be elucidated and the dose‐effect relationship, if any, is yet to be described. We believe that our results should be taken into consideration in the evaluation of the risk‐benefit balance of childhood GH treatments.

## CONFLICT OF INTEREST

A. Poidvin received a master grant from the French Endocrine Society (Société Française d'Endocrinologie) funded by Lilly. J.C. Carel has the following conflicts of interest to declare, all outside the scope of this work: investigator in clinical trials using GH sponsored by Pfizer and Lilly and in postmarketing studies using several brands of GH, and support for travel to international meetings from several GH manufacturers. Emmanuel Ecosse, Dominique Levy, Jean Michon, and Joël Coste report no disclosures relevant to the manuscript.
