# Supplementary material for: Increased risk of bone tumors after growth hormone treatment in childhood: A population‐based cohort study in France
Source: Cancer Med. 2018 Jun 14;7(7):3465–73. doi: 10.1002/cam4.1602 (PMC6051149; doi:10.1002/cam4.1602)
Supplement: Supplementary file 1 [file CAM4-7-3465-s001.docx]

| **Supplementary Table 1.** Eight log-linear models for three sources capture-recapture analyses. with the Akaike Criterion and estimated number of missing cases | | |
| --- | --- | --- |
| **Model** | **Akaike Criterion** | **Cases missed (95% CI)** |
| *Independent* | 30.6 | 1.8 (0.4-7.0) |
| *Questionnaire-LLA* | 31.2 | 0.4 (0.01-20.5) |
| ***LLA-FHDD **** | **27.9** | **3.5 (0.8-15.3)** |
| *FHDD-Questionnaire* | 32.3 | 1.2 (0.2-9.4) |
| *Questionnaire-LLA; LLA-FHDD* | 29.6 | 1.2 (0.02-98.5) |
| *Questionnaire-LLA; FHDD-Questionnaire* | 32.2 | 0.1 (0.01-12.3) |
| *LLA-FHDD; FHDD-Questionnaire* | 29.9 | 3.5 (0.3-35.5) |
| *LLA-FHDD; FHDD-Questionnaire; Questionnaire-LLA* | 31.5 | 1.0 (0.01-175.9) |
|  |  |  |
| LLA: long-lasting affection. FHDD: French hospital discharge database  * the chosen model. - : source dependence. 95% CI: 95% Confidence intervals | | |
